# Which aspects of health differ between working and nonworking women with fibromyalgia? A cross-sectional study of work status and health

**DOI:** 10.1186/1471-2458-12-1076

**Published:** 2012-12-14

**Authors:** Annie Palstam, Jan L Bjersing, Kaisa Mannerkorpi

**Affiliations:** 1 Department of Rheumatology and Inflammation Research, Institute of Medicine, Sahlgrenska Academy, University of Gothenburg, Gothenburg, Sweden; 2 Rheumatology, Sahlgrenska University Hospital, Gothenburg, Sweden; 3 Physiotherapy and Occupational Therapy, Sahlgrenska University Hospital, Gothenburg, Sweden; 4 Sahlgrenska Academy, University of Gothenburg Centre for Person-centred Care (GPCC), Gothenburg, Sweden

**Keywords:** Fibromyalgia, Work, Health, Women, Physical, Pain

## Abstract

**Background:**

Women with fibromyalgia (FM) describe great difficulties in managing work. Reported work ability in women with FM varies from 34 to 77 percent in studies from different countries. Many factors are suggested to affect the ability to work in women with FM, including pain, fatigue, impaired physical capacity and activity limitations. However, it is difficult to define to which extent symptom severity can be compatible with work. The aim of this study was to investigate which aspects of health differ between working women with FM and nonworking women with FM.

**Methods:**

A cross-sectional study of 129 women of working age with FM which included clinical assessment, structured interviews, questionnaires and performance-based tests. The women were categorized as working or nonworking. Aspects of health are presented according to the International Classification of Functioning, Disability and Health (ICF).

**Results:**

Working women with FM presented better health than nonworking women with FM in ratings of body function (FIQ pain *p* < 0.001, FIQ fatigue *p* = 0.006, FIQ stiffness *p* = 0.009, HADS-Depression *p* = 0.007). Ratings of overall health status were also significantly better in working women with FM than in nonworking women with FM (FIQ total, eight-item *p* = 0.001 and SF-36 PCS *p* < 0.001). No significant differences were found between working- and nonworking women in tests of physical capacity. FIQ pain was an independent explanatory factor for work in stepwise multiple logistic regression analysis (OR 0.95, CI 0.93- 0.98), *p* < 0.001.

**Conclusion:**

Working women with FM reported better health than nonworking women with FM in terms of pain, fatigue, stiffness, depression, disease specific health status and physical aspects of quality of life, which represent body functions and overall health status. However, they were equally impaired in tests of physical capacity. Moderate pain levels were compatible with work, while severe pain appeared to compromise work. Fatigue was better tolerated, as women scoring severe levels of fatigue worked.

## Background

The research criteria of fibromyalgia (FM) as defined by the American College of Rheumatology (ACR) criteria for FM include a history of widespread pain for at least three months and pain on manual palpation in 11 of 18 tender points [1]. FM is characterized by persistent widespread pain, increased pain sensitivity and tenderness [1]. Other associated symptoms are fatigue, psychological distress [1,2], activity limitations [3] and impaired physical capacity [4]. The prevalence of FM ranges from 1 to 3% in the general population, it is more common among women and increases with age [2,5].

Activity limitations in FM have an impact on work ability [6]. FM imposes a heavy patient burden in terms of disability, loss of quality of life and costs, and it imposes an economic burden on society [7]. The degree of employment in FM varies geographically, with a range from 34% to 77% in different studies [8]. The wide range is related to differences in the social benefit systems and labour markets of different countries [8]. Working women with FM have previously been reported to experience less pain, less fatigue and better functional status than nonworking women with FM [9]. Severe pain and fatigue combined with a demanding life situation and ageing have been associated with work disability in FM, as well as self rated disability and unmarried status [10,11].

Disability benefits in Sweden are approved when a disease impairs a person’s ability to work by at least 25%. Approximately 72% of all women in Sweden of working age (16–64 years) were employed in the year 2005 and 8% received full-time disability benefits_1_.

Interview studies have indicated that the severity of symptoms and psychosocial and environmental factors influence work disability in women with FM [3,8,11-14]. These findings are supported by results of surveys conducted in large populations [6,10]. However, it is difficult to define to which extent symptom severity can be compatible with work. Assessments of physical, social and psychological health components combining subjective ratings with performance-based tests would advance our understanding in this area.

### Objective

The purpose of this study was to investigate which aspects of health differ between working women (WW) with FM and nonworking women (NWW) with FM. We hypothesized that WW with FM would display better health than NWW with FM in terms of subjective ratings of health and performance-based tests of physical capacity.

## Methods

### Study design

A cross -sectional study of work status and health in women with FM.

### Participants

Women with FM were recruited to an experimental study [14] from three primary health-care centres in West Sweden by systematic search of patient journals and by consecutive recruitment. The inclusion criteria were women who were 18–60 years of age and suffered FM according to the American College of Rheumatology (ACR) criteria for FM [1]. The search of patient journals found 298 potentially eligible women who were contacted by post (n = 55) or telephone (n = 243) for further screening. Forty-eight women could not be contacted, 55 did not meet inclusion criteria at telephone screening, and 61 declined to participate in the study, while 134 agreed to participate in an examination. Twenty-three of them did not meet inclusion criteria at examination, 17 were excluded due to; treatment in progress (n = 3), severe disorders (n = 9) or unemployment (n = 5) and 18 declined to participate. At the same time, 93 women were consecutively recruited to the study. Fifty-three of them fulfilled inclusion criteria and agreed to participate in the study. A total of 129 women with FM formed the study population. The study population was divided into two groups according to work status; WW (25-100%) and NWW (0%). The WW included 13 full-time workers (80-100%), 13 part-time workers working less than 50% (25-49%), 17 part-time workers working 50%, and 10 part-time workers working 50% or more (50-75%). Thirty-seven part-time working women received disability benefits while three did not. All NWW received disability benefits.

### Data collection

Clinical assessments of tender points by manual palpation [1] and muscle tenderness with the Somedic Algometer (Somedic Production AB, Sollentuna, Sweden) [15] were conducted by trained examiners to verify diagnosis according to the American College of Rheumatology criteria for FM [1]. Demographic data including work status were gathered in a standardized interview. The women completed a battery of questionnaires and performed two tests of physical capacity described in detail below. Aspects of health are presented according to the International Classification of Function, Disability and Health (ICF) [16].

### Measures

#### Personal- and environmental factors

Information on age, employment, educational level, cohabitation, ethnicity and brief medical history was obtained in a standardized interview. Mean household income per geographical area was based on zip codes and obtained by Statistics Sweden_1_.

The *Fibromyalgia Impact Questionnaire* (*FIQ*) is disease specific and comprises ten subscales of disabilities and symptoms ranging from 0 to 100. A higher score indicates a lower health status [17]. The subscale *FIQ feel good* is presented as a personal factor according to the ICF [18].

*Medical Outcome Study* - *Social Support Survey fouritem scale* (*MOSSSS*) is a short version of the 18-item MOS-SSS consisting of a four-item social support scale (1–5) covering four different categories of social support: emotional, tangible, affectionate and positive social interaction. The total score ranges from four to 20. A higher score indicates a higher degree of perceived social support [19].

#### Body function

The s*ixminute walk test* (*6MWT*) is a performance-based test that measures total walking distance during a period of six minutes [20,21]. The 6MWT is considered a useful representation of physical capacity and endurance in daily life [22].

*Grippit* (*AB Detektor*, *Göteborg*, *Sweden*) is an electronic instrument that measures hand grip force. The mean force over a set period of time (ten seconds) was recorded [20].

*Pain localization*, this is a self administered sheet that records the number of pain localizations (0–18), based on a drawing of the body with 18 predefined regions [23].

The *Fibromyalgia Impact Questionnaire* (*FIQ*) is disease specific and comprises ten subscales of disabilities and symptoms ranging from 0 to 100. A higher score indicates a lower health status [17]. The subscales *FIQ pain*, *FIQ fatigue*, *FIQ morning tired*, *FIQ stiffness*, *FIQ anxiety*, *and FIQ depression* are presented as factors of body function according to the ICF [18].

*Hospital Anxiety and Depression Scale* (*HADS*) contains 14 statements, ranging from 0 to 3, in which a higher score indicates a higher degree of distress. The scores build two subscales: HADS-A for anxiety (0–21) and HADS-D (0–21) for depression. The cut-off score of eight is suggested to indicate possible anxiety and depression [24]. This scale is categorized as a measure of body function since 93% of its content concerns this ICF domain [18].

*Multidimensional Fatigue Inventory* (*MFI20*) contains 20 statements that build five subscales. Each subscale ranges from four to 20 and a higher score indicates a higher degree of fatigue [25,26]. This inventory is categorized as a measure of body function since 67% of its content concerns this ICF domain [18].

#### Activity and participation

The *Leisure Time Physical Activity Instrument* (*LTPAI*) is a questionnaire that assesses the amount of time spent on physical activity during a typical week. The total score is the sum of hours of activities [27].

The *Fibromyalgia Impact Questionnaire* (*FIQ*) is disease specific and comprises ten subscales of disabilities and symptoms ranging from 0 to 100. A higher score indicates a lower health status [17]. The subscale *FIQ physical function* is presented as a factor of activity and participation according to the ICF [18].

#### Health status – elements in overall health status

The *Fibromyalgia Impact Questionnaire* (*FIQ*) is disease specific and comprises ten subscales of disabilities and symptoms ranging from 0 to 100. The total score is the mean of ten subscales. A higher score indicates a lower health status [17]. Two subscales of the FIQ total score were omitted here (Work missed and Job ability); thus an eight-item total score of the FIQ was applied in the study. FIQ subscales are presented according to ICF [18].

*ShortForm 36* (*SF36*) is a generic questionnaire that assesses health related quality of life, comprising eight subscales ranging from 0 to 100. A higher score indicates a better quality of life [28]. The subscales that build two composite scores, the Physical Component Scale (PCS) and the Mental Component Scale (MCS), were used in this study and are presented as health status.

### Statistical analysis

Descriptive data are presented as mean and standard deviation (SD), median and range or the number (N) and percent. The Mann- Whitney U-test was used for analyses of between-group differences in continuous variables. The Mantel Haenzel test and Fisher’s exact test were used for analyses of between-group differences in ordinal categorical variables. P-values of ≤0.010 were considered significant. To control possible Type I errors, the upper limit of expected number of false significances for the analyses was calculated by the following formula: α/1– α × (number of tests – number of significant tests), where α is the significance level. Variables displaying statistically significant differences in between-group analyses of WW and NWW were included in stepwise multiple logistic regression analysis to evaluate explanatory factors for work. The order of inclusion was based on the level of significance of each variable, where the variable presenting the highest level of significance was included first in the model. An odds-ratio (OR) with 95% CI is presented for descriptive purposes. The area under the ROC curve (AUC statistics) was calculated for a description of the goodness of explanatory variables.

### Ethics

The study was approved by the ethics committee at the Sahlgrenska Academy, University of Gothenburg. Written and oral informed consent was obtained from all participants.

## Results

### Study population

The mean age was 45.7 years (SD 8.7). The mean duration of symptoms was 10.5 years (SD 7.1). The mean number of tender points was 14.8 (SD 2.4) and the mean pain threshold was 171 kPa/sec (SD 66). There were no significant differences in pain threshold or the number of tender points between WW and NWW.

### Type I error

The between-group analyses comprised a total of 33 statistical analyses, with 11 significant values at significance level 0.01, and the upper level of number of false significances was 0.2, which indicates that 0–1 of the significances found might be false.

### Personal- and environmental factors

#### Personal factors

No significant differences were found between WW and NWW in personal factors.

#### Environmental factors

No significant differences were found between WW and NWW in environmental factors (see Table 1).

**Table 1:** Personal- and environmental factors in working women (WW) and nonworking women (NWW) with fibromyalgia

	**WW (N = 53)**		**NWW (N = 76)**		***p*-*value***
*Personal factors*	*Mean (SD)*	*Median (range)*	*Mean (SD)*	*Median (range)*	
Age, *years*	45.4 (8.1)	47 (22–57)	46.0 (9.2)	47 (24–60)	0.567
Symptom duration, *years*	11.7 (5.8)	10 (2–24)	9.7 (7.9)	8 (0.3-45)	0.021
FIQ feel good, *0-100*	69.0 (28.9)	71 (0–100)	81.3 (22.7)	86 (0–100)	0.014
	*N (%)*		*N (%)*		
Living with an adult	45 (84.9)		55 (72.4)		0.133
Born outside of Sweden	8 (15.1)		13 (17.1)		0.813
Education:					
≤ 9 years	11 (20.8)		17 (22.7)		
10 – 12 years	29 (54.7)		40 (53.3)		
>12 years	13 (24.5)		18 (24.0)		0.843
Pharmacological treatment:					
Analgesic/NSAID, *yes*	31 (58.5)		58 (76.3)		0.035
Psychotropics, *yes*	22(41.5)		37 (48.7)		0.475
*Environmental factors*	*Mean (SD)*	*Median (range)*	*Mean (SD)*	*Median (range)*	
MOS-SSS, *4-20*	15.4 (4.0)	16 (7–20)	14.5 (4.1)	15 (4–20)	0.267
Mean income in area of residence, *1000 Swedish kronor*	214 (30.1)	214 (165–299)	201 (29.7)	205 (123–267)	0.042

P-values of ≤0.01 are considered significant and shown in bold type.

FIQ: Fibromyalgia Impact Questionnaire, MOS-SSS: Medical Outcome Study –Social Support Survey 4-item scale.

Missing values: FIQ feel good (n = 3), education (n = 1), mean income in the area of residence (n = 6).

### Body function

#### Performance based tests of physical capacity

No significant differences were found between WW and NWW.

#### Self rated body function

The number of pain localizations was fewer in WW than in NWW (*p* = 0.009) and pain (FIQ pain) was milder in WW than in NWW (*p* < 0.001). Stiffness (FIQ stiffness) was milder in WW than in NWW (*p* = 0.009). Fatigue was less severe in WW than in NWW (FIQ fatigue *p* = 0.006, MFI physical fatigue *p* = 0.001, MFI reduced activity *p* = 0.001 and MFI mental fatigue *p* = 0.006). WW rated a lower level of depression (HADS-D) than NWW (*p* = 0.007). Fifty-two percent of NWW and 29% of WW scored above the cut-off score for possible depression. There was no significant difference in anxiety (HADS-A) between WW and NWW. Fifty-eight percent of the NWW and 47% of the WW scored above the cut-off score for possible anxiety (8).

### Activity and participation

No significant differences were found between WW and NWW in leisure time physical activity (LTPAI) or activity limitations in daily life (FIQ physical function).

### Health status

A better disease specific health status (FIQ total, eight-item) was found in WW than in NWW (*p* = 0.001). This was also true for physical health related quality of life (SF-36 PCS) (*p* < 0.001) (see Table 2).

**Table 2:** Body function, activity and health status in working women (WW) and nonworking women (NWW) with fibromyalgia

	**WW (N = 53)**		**NWW (N = 76)**		***p-value***
*Body function,*	*Mean (SD)*	*Median (range)*	*Mean (SD)*	*Median (range)*	
*Performance-based tests*					
6MWT, *meters*	520 (95.6)	524 (136–674)	500 (75.8)	512 (295–686)	0.087
Grippit right hand, *Newton*	160.3 (67.1)	155 (27–323)	146.1 (67.6)	160 (13–334)	0.284
Grippit left hand, *Newton*	158.6 (73.2)	155(17–349)	144.3 (65.4)	147 (19–319)	0.343
*Body function, ratings*					
Pain localizations, *number*	12.5 (3.3)	13 (5–18)	14.0 (3.2)	14 (5–18)	**0.009**
FIQ pain, *0-100*	62.5 (17.1)	63 (26–100)	77.0 (17.0)	80 (26–100)	**<0.001**
FIQ fatigue, *0-100*	75.4 (22.4)	83 (15–100)	84.8 (17.1)	90 (19–100)	**0.006**
FIQ morning tired, *0-100*	76.9 (20.7)	81 (10–100)	83.6 (18.9)	89 (2–100)	0.017
FIQ stiffness, *0-100*	64.0 (27.6)	75 (15–97)	75.6 (23.7)	82 (10–100)	**0.009**
FIQ anxiety, *0-100*	42.1 (32.6)	38 (0–96)	52.5 (35.1)	54 (0–100)	0.060
FIQ depression, *0-100*	39.2 (31.7)	32 (0–96)	48.5 (32.5)	51 (0–100)	0.116
HADS-A, *0-21*	7.5 (4.8)	7 (1–19)	9.7 (5.2)	9 (1–20)	0.021
HADS-D, *0-21*	6.2 (2.9)	6 (2–15)	8.0 (3.9)	8 (1–16)	**0.007**
MFI General Fatigue, *4-20*	16.8 (3.0)	18 (9–20)	18.0 (2.4)	19 (12–20)	0.021
MFI Physical Fatigue, *4-20*	16.2 (3.2)	17 (9–20)	18.0 (2.2)	19 (10–20)	**0.001**
MFI Reduced Activity, *4-20*	14.5 (3.3)	14 (8–20)	16.5 (3.5)	17 (7–20)	**0.001**
MFI Reduced Motivation, *4-20*	9.7 (3.1)	10 (5–16)	10.9 (4.3)	11 (4–19)	0.123
MFI Mental Fatigue, *4-20*	13.5 (3.5)	14 (5–20)	15.1 (4.0)	16 (4–20)	**0.006**
*Activity and participation*					
LTPAI, *hours*	4.5 (3.9)	3 (1–23)	5.4 (3.7)	4 (1–18)	0.088
FIQ physical function, *0-100*	39.8 (20.5)	40 (3–90)	49.9 (23.1)	53 (0–100)	0.013
*Health Status*					
FIQ total, 8-item, *0-100*	58.7 (17.1)	63.5 (16–88)	69.3 (14.5)	71.3 (25–95)	**0.001**
SF-36 PCS, *0-100*	32.6 (8.0)	33.3 (15–48)	27.0 (7.0)	27.6 (11–46)	**<0.001**
SF-36 MCS, *0-100*	41.6 (12.7)	43 (17–68)	36.8 (13.4)	37.5 (16–64)	0.043

P-values of ≤0.01 are considered significant and shown in bold type.

6MWT: six-minute walk test; FIQ: Fibromyalgia Impact Questionnaire; MFI: Multidimensional Fatigue Inventory; HADS-A and -D: Hospital Anxiety and Depression Scale for - anxiety and - depression; LTPAI: Leisure Time Physical Activity Instrument; SF-36 PCS and MCS: Short Form −36, Physical Component Scale and Mental Component Scale.

Missing values: Grippit (n = 1), FIQ Pain (n = 1), FIQ depression (n = 1), FIQ physical function (n = 2), LTPAI (n = 1), 8-item FIQ total (8) (n = 7), SF-36 (n = 3).

### Stepwise multiple logistic regression analyses

Variables displaying a significant difference (*p* ≤ 0.010) between WW with FM (n = 53) and NWW with FM (n = 76) were included in stepwise multiple logistic regression analysis. FIQ pain (n = 128) was the only statistically significant variable to independently explain work (OR 0.95, CI 0.93- 0.98), *p* < 0.001, (AUC 0.75, CI 0.66- 0.83).

## Discussion

The main finding in this study was that working women (WW) with FM displayed better ratings than nonworking women (NWW) with FM in terms of pain, fatigue, stiffness, depression, disease specific health status and physical health related quality of life, which represent body functions and overall health status.

Physical capacity did not differ significantly between WW and NWW in terms of performance-based tests (see Table 2) where both groups presented lower capacity than the average population [20,21]. This supports earlier studies showing impaired body function in women with FM [4,29]. However, the physical work demands might influence the work ability in persons who have an impaired physical capacity. Earlier studies have reported the importance of the work environment in women with FM [8,11,13,30-32] and in other rheumatic diseases [33].

The number of pain localizations was significantly lower in WW than in NWW and global pain (FIQ pain) was significantly milder in WW than in NWW (see Table 2). The mean pain (FIQ pain) of WW was well above 50 (0–100), which corresponds to the average pain level in previous studies of FM [34]. Mean pain was above 75 (0–100) in NWW, which corresponds to the ratings of severely afflicted patients with FM [34]. FIQ pain was found to be the only independent explanatory factor for work in this study. Pain has previously been found to be a critical factor for work in rheumatic diseases [10,35]. Our results indicate that women with FM having moderate pain generally could be expected to work. Some women appear to be able to work despite severe pain, which raises the question if there are workplace related factors that support their ability to work [32,36]. The influence of work related factors on work ability in FM need to be further studied.

Global fatigue (FIQ fatigue) was found to be significantly lower in WW than in NWW as well as physical fatigue (MFI-20), reduced activity (MFI-20), and mental fatigue (MFI-20) (see Table 2). Fatigue has previously been found to be an important factor for work disability in rheumatic diseases [35]. However, our results showed severe global fatigue (FIQ fatigue) with mean ratings of over 70 (0–100) [34] also in WW, indicating that fatigue might not be a critical factor for work disability.

Depression was rated significantly lower in WW than in NWW in the HADS, assessing depression. This supports the results of an earlier study on work disability in FM reporting the negative impact of depression symptoms on work ability [37].

WW displayed a significantly better disease specific health status (FIQ total, eight-item) than NWW (see Table 2). This supports the results from an earlier study on work disability in FM where the FIQ total score was found to predict work disability [6]. Physical health-related quality of life (SF-36 PCS) was significantly higher in WW than in NWW (see Table 2), which is in line with a previous study of FM [38]. However, the quality of life of workers in our population, assessed by SF-36, was very low as compared to a national sample [39]. Impaired health status assessed by SF-36 has earlier been associated with work disability in rheumatoid arthritis (RA) [35], systemic lupus erythematosus (SLE) [40] and musculoskeletal pain [41].

The theory of the healthy worker effect suggests that healthier individuals are more likely to remain in the workforce [42]. On one hand, this agrees well with the results of the present study. On the other hand, work is an important factor for health status in women in general [43,44] and in women with FM [38]. Further studies are needed to explore if working women with FM maintain their health status, or if it deteriorates over time.

The main strength of the present study is the integration of physical, social and psychological assessments including subjective ratings as well as clinical assessments and performance-based tests of physical capacity. About 40% of the patients in this study worked part-time or full-time which is in line with international reports of work ability in FM [8]. No significant differences were found in age, symptom duration, cohabitation, ethnicity, education, pharmacological treatment, mean income in the area of residence and social support, i.e. personal and environmental barriers or facilitators for health [16]. A limitation of this study is the cross sectional design which does not allow analyses of cause and effect. Also, the specific demands in work were not reported in the study and need further investigation.

## Conclusions

Working women with FM reported better health than nonworking women with FM in terms of pain, fatigue, stiffness, depression, disease specific health status and physical aspects of quality of life, which represent body functions and overall health status. However, they were equally impaired in tests of physical capacity. Moderate pain levels were compatible with work, while severe pain appeared to compromise work. Fatigue was better tolerated, as women scoring severe levels of fatigue worked.

## Endnotes

^a^Statistics, Sweden. Stockholm: SCB 2008; 2011 [updated 2011 May 16] Available from: http://www.scb.se.

## Competing interests

The authors declare that they have no competing interests.

## Authors’ contributions

AP participated in the design of the study, performed the statistical analysis and drafted the manuscript. JB participated in the design of the study and helped to draft the manuscript. KM conceived of the study, and participated in its design and helped to draft the manuscript. All authors read and approved the final manuscript.

## Pre-publication history

The pre-publication history for this paper can be accessed here:

http://www.biomedcentral.com/1471-2458/12/1076/prepub
